# Supplementary material for: HIVprotI: an integrated web based platform for prediction and design of HIV proteins inhibitors
Source: J Cheminform. 2018 Mar 9;10:12. doi: 10.1186/s13321-018-0266-y (PMC5845081; doi:10.1186/s13321-018-0266-y)
Supplement: Supplementary file 2 — Additional file 2. Source code of HIVProtI web server. [file 13321_2018_266_MOESM2_ESM.zip › HIVprotI_Source-code/hivprotI/weka-3-6-6/doc/weka/gui/treevisualizer/PlaceNode1.html]

PlaceNode1


---


|  |  |  |
| --- | --- | --- |
| **Overview** \| **Package** \| **Class** \| **Tree** \| **Deprecated** \| **Index** \| **Help** \| **Weka's home** | |  |
| **PREV CLASS**   **NEXT CLASS** | **FRAMES**    **NO FRAMES**     **All Classes** |  |
| SUMMARY: NESTED \| FIELD \| CONSTR \| METHOD | DETAIL: FIELD \| CONSTR \| METHOD |  |


---


## weka.gui.treevisualizer Class PlaceNode1

```
java.lang.Object
  weka.gui.treevisualizer.PlaceNode1
```

**All Implemented Interfaces:**: NodePlace

---

``` public class PlaceNode1 extends java.lang.Object implements NodePlace ```

This class will place the Nodes of a tree.

It will place these nodes so that they symetrically fill each row.
This is simple to calculate but is not visually nice for most trees.

**Version:**
:   $Revision: 1.4 $

**Author:**
:   Malcolm F Ware (mfw4@cs.waikato.ac.nz)

---

**Constructor Summary**

`PlaceNode1()`


| **Method Summary** | |
| --- | --- |
| `void` | `place(Node r)`             Call this function to have each node in the tree starting at 'r' placed in a visual (not logical, they already are) tree position. |

**Methods inherited from class java.lang.Object**

`equals, getClass, hashCode, notify, notifyAll, toString, wait, wait, wait`

**Constructor Detail**

### PlaceNode1

```
public PlaceNode1()
```


**Method Detail**

### place

```
public void place(Node r)
```

:   Call this function to have each node in the tree starting at 'r' placed
    in a visual
    (not logical, they already are) tree position.

    :   **Specified by:**: `place` in interface `NodePlace`
    :   **Parameters:**: `r` - The top of the tree.


---


|  |  |  |
| --- | --- | --- |
| **Overview** \| **Package** \| **Class** \| **Tree** \| **Deprecated** \| **Index** \| **Help** \| **Weka's home** | |  |
| **PREV CLASS**   **NEXT CLASS** | **FRAMES**    **NO FRAMES**     **All Classes** |  |
| SUMMARY: NESTED \| FIELD \| CONSTR \| METHOD | DETAIL: FIELD \| CONSTR \| METHOD |  |


---
